# Development and Validation of a Clinical Decision Support Tool to Predict Disease Progression in Crohn’s Disease Treated with Ustekinumab

**DOI:** 10.3390/jcm14227919

**Published:** 2025-11-08

**Authors:** Lingya Yao, Yushu Cao, Chenhao Bai, Rongbei Liu, Wenjing Yang, Kang Chao, Zhaopeng Huang, Yun Qiu, Xiang Gao, Minhu Chen, Qian Cao

**Affiliations:** 1 Department of Gastroenterology, Sir Run Run Shaw Hospital, Zhejiang University School of Medicine, Hangzhou 310016, China; 11918256@zju.edu.cn (L.Y.); 12318714@zju.edu.cn (Y.C.); bch@zju.edu.cn (C.B.); liurongbei@zju.edu.cn (R.L.); yangwenjing@zju.edu.cn (W.Y.); 2 Inflammatory Bowel Disease Center, Sir Run Run Shaw Hospital, Zhejiang University School of Medicine, Hangzhou 310016, China; 3 Department of Gastroenterology, The Sixth Affiliated Hospital of Sun Yat-sen University, Guangzhou 510655, China; chaokang3@mail.sysu.edu.cn (K.C.); huangzhp23@mail2.sysu.edu.cn (Z.H.); gxiang@mail.sysu.edu.cn (X.G.); 4 Department of Gastroenterology, The First Affiliated Hospital of Sun Yat-sen University, Guangzhou 510080, China; qyun@mail2.sysu.edu.cn (Y.Q.); chenminhu@mail.sysu.edu.cn (M.C.)

**Keywords:** Crohn’s disease, ustekinumab, clinical decision support tool

## Abstract

**Background/Objectives:** Ustekinumab (UST) is an effective and safe drug for treating Crohn’s disease (CD), but data on disease progression after UST treatment is limited. This study aimed to develop a clinical decision support tool (CDST) to identify Chinese patients with CD less likely to experience disease progression during UST treatment. **Methods**: A multicenter, retrospective observational study was conducted among Chinese patients with CD who started UST treatment between 1 May 2020 and 20 October 2022. Baseline characteristics, defined as the measurements taken closest to, and prior to, the first dose of UST, were collected. Disease progression, defined as CD-related surgery, hospitalization, and complications, was evaluated by week 52 (±4 weeks). Predictors were identified using logistic regression, and a UST-specific CDST (UST-CDST) was developed. The UST-CDST was then internally and externally validated using the area under the receiver operating characteristic curve (AUC). **Results**: Among 602 enrolled patients, 533 were included in the analysis. Four factors were suggestively associated with disease progression: prior biologics usage, baseline disease severity, baseline C-reactive protein, and baseline hemoglobin. The prediction model demonstrated an AUC of 0.88 in internal validation and 0.66 in external validation. The UST-CDST effectively stratified patients into low-risk or high-risk groups for disease progression within one year. **Conclusions**: A UST-CDST was developed and validated to identify patients with CD less or more likely to experience disease progression around week 52 under UST therapy. The scoring system promises to facilitate clinical decision-making and personalized treatment.

## 1. Introduction

Crohn’s disease (CD), a major subtype of inflammatory bowel disease (IBD), is a chronic progressive inflammatory disorder requiring long-term therapy [1,2,3]. Owing to rapid urbanization, IBD has become a more common condition in China, with a prevalence of approximately 10 cases per 100,000 population, causing a substantial burden on medical care [2,4]. In recent years, biologics have been widely used in the management of moderate to severe CD, including anti-tumor necrosis factor (anti-TNF)-α agents, vedolizumab (VDZ) and ustekinumab (UST) [5]. UST, a monoclonal antibody against the p40 subunit of interleukin-12 and interleukin-23, is particularly recommended for patients with CD who have not responded to anti-TNF therapy [6]. Since its approval in China in 2020 for moderate to severe CD, UST has been increasingly adopted in clinical practice [7]. A recent real-world study involving 5077 Chinese patients with CD revealed that 161 (3.2%) were treated with UST, reflecting its growing utilization in the post-approval era [8]. The long-term efficacy and safety of UST were characterized in several clinical trials, including UNITI-1, UNITI-2, and IM-UNITI [3,6,9]. However, long-term data for patients with complex disease phenotypes and those who have failed biologic therapy are still lacking [10]. Furthermore, 30% to 40% of patients with CD who initially responded to UST experienced a loss of therapeutic effect during these clinical trials [9,11].

Personalized treatment is increasingly valued and will become essential in IBD management [12]. With the emergence of new therapeutic options for IBD, there is an urgent but unmet need for predicting response before treatment initiation. This would reduce health care costs, avoid unnecessary treatment, and allow more rational use of social and medical resources [13]. Currently, several factors hold potential for predicting the response to biologics, including disease behavior, disease severity, laboratory measures (e.g., C-reactive protein, albumin), prior drug usage, proteomic biomarkers, and certain colorectal mucosa features [14]. The development of clinical prediction models integrating multiple patients’ characteristics may help to predict drug efficacy and safety [15]. Dulai et al. developed a clinical decision supporting tool (CDST) to predict clinical remission by week 16 of UST therapy in patients with active CD from the UNITI trials [6,16]. The CDST was calculated using the following baseline factors: albumin, smoking history, prior bowel surgery, actively draining fistula, and prior anti-TNF-α exposure [16]. Moreover, the UNITI-derived CDST was demonstrated to predict clinical relapse (defined as corticosteroid addition, UST withdrawal, CD-related surgery, and hospitalization during UST maintenance therapy) with an area under the curve (AUC) of 0.698 (95% CI, 0.588–0.807) among 99 Korean patients with moderate to severe CD during a median follow-up period of 18 months [17]. However, the relatively small sample size of the Korean cohort has limited confidence in supporting the generalizability of the UNITI-derived CDST to Asian populations. Additionally, no CDST models have been specifically designed for predicting disease progression in patients receiving UST therapy.

Existing CDSTs are often limited by small sample size or have not been specifically developed and validated for the unique characteristics of the Chinese CD population. Therefore, the primary objective of this study was to develop and validate a CDST specifically designed to predict the risk of long-term disease progression in Chinese patients with CD undergoing UST therapy. External validation was performed to confirm the clinical utility of the UST-CDST. The pre-assessment of UST efficacy can facilitate clinical decision-making process to select more appropriate biological agents for individual patients [18].

## 2. Materials and Methods

### 2.1. Study Design

The retrospective, observational, multicenter study was conducted in three referral hospitals in China. Patients with CD were enrolled and followed up for 52 ± 4 weeks if they initiated UST from 1 May 2020 to 20 October 2022. We developed and validated a multivariable model to predict disease progression in patients treated with UST. The study complied with the Transparent Reporting of a Multivariable Prediction Model for Individual Prognosis or Diagnosis (TRIPOD) statement and the Strengthening the Reporting of Observational Studies in Epidemiology (STROBE) statement. The study has been registered in the Chinese Clinical Trial Registry (Registration number: ChiCTR2200067162) and approved by the Ethical Review Committee of the Sir Run Run Shaw Hospital, Zhejiang University School of Medicine (Ethical number: 2022-0463).

### 2.2. Study Population

Data were collected from two hospital-based cohorts (Cohort I and Cohort II) to establish and validate a clinical prediction model. Cohort I was used for model training and internal validation, while Cohort II was used for external validation.

Cohort I was derived from the Sir Run Run Shaw Hospital IBD Biobank in China (SRRSH-IBC). Information on patients with admission records from 1999 and 2020 was retrospectively collected and verified by experienced IBD clinicians before entry into the SRRSH-IBC database. From 2020 onwards, SRRSH-IBC began prospectively recruiting patients with IBD during their visits to SRRSH. All patients were prospectively followed up at intervals of every 3 to 6 months. By the end of June 2024, a total of 10,690 patients with IBD had been enrolled in SRRSH-IBC. A wide range of data on demographical and disease characteristics were collected at baseline, including sex, age at symptom onset, age at diagnosis, family history of IBD, history of medical and surgical treatment before diagnosis, disease location, disease behavior, perianal involvement, extra-intestinal manifestations (EIMs), pathology reports, endoscopy reports, radiologic features, and lifestyle information. Biological samples, including blood, stool, and biopsied tissues, were collected at baseline and at certain time points during follow-up. Cohort II was derived from the First Affiliated Hospital and the Sixth Affiliated Hospital of Sun Yat-sen University. The data from these hospitals were retrospectively collected from the medical record system by well-trained IBD researchers. Diagnosis and treatment strategies of IBD in the above three centers all complied with the European Crohn’s and Colitis Organization (ECCO) guideline [19].

In this study, the inclusion criteria were defined as follows: (1) Patients with a confirmed diagnosis of CD; (2) patients who initiated UST between 1 May 2020 and 20 October 2022; (3) patients followed up for at least 1 year after UST initiation. The exclusion criteria were as follows: (1) Patients previously received UST for other clinical indications; (2) patients with incomplete medical records that prevented the calculation of the CDST (detailed below).

### 2.3. Predicting Variables

We collected and examined the following data from the SRRSH-IBC or the electronic medical records of patients: sex, age at diagnosis, age at first UST treatment, body mass index, smoking status, C-reactive protein (CRP), erythrocyte sedimentation rate, white blood cell count, neutrophil count, blood platelet count, hemoglobin, albumin level, concomitant drug usage (referring to a drug taken by the patient concurrently with UST but that does not influence the efficacy of UST), history of perianal surgery, history of gastrointestinal surgery, prior steroid usage, prior immunosuppressant usage, prior biologics usage, EIMs, perianal involvement, disease behavior and location (according to the Montreal classification [20]), disease severity (Crohn’s Disease Activity Index [CDAI], assessed by specialized nurses during each hospital admission), and disease duration. All baseline data were defined as the measurements taken closest to, and prior to, the first dose of UST.

### 2.4. Clinical Outcomes and Definitions

The outcome was disease progression at week 52 (±4 weeks) after UST treatment. The definition of disease progression referred to both STRIDE-II consensus on treat-to-target strategies and Chinese consensus [21,22]. The time point of assessment was chosen based on clinical practice in China and previous clinical trials of UST [9,22]. Disease progression was defined as the occurrence of any of the following conditions according to IBD management guidelines [20]. The first condition was CD-related surgery, including bowel resection surgery, anastomosis, abdominal abscess drainage, stenosis/guided endoscopic balloon dilatation, colostomy, and perianal surgery (drainage of perianal abscess and fistula seton). The second condition was CD-related hospitalization, which was defined as hospitalization for CD-related surgery, CD-related complications, or treatment adjustment. Hospitalization for routine endoscopic evaluation or regular drug injection was not included. The third condition was CD-related complications, including the development of intestinal penetration or strictures and perianal fistulas or abscesses.

### 2.5. Statistical Analysis

#### 2.5.1. Handling of Missing Data

Predictor variables with >10% missing values were excluded from the modeling process to ensure robustness. For the remaining dataset, we performed multiple imputation using the Multivariate Imputation by Chained Equations (MICE) package in R with predictive mean matching, generating 5 imputed datasets. The imputation model included all variables intended for the subsequent univariate and multivariate analyses. After imputation, models were built on each dataset, and the results were pooled according to Rubin’s rules. 21 patients with missing data on key variables for the final model were excluded.

#### 2.5.2. Random Split Verification

Cohort I was randomly divided into training and validation sets with a ratio of 7:3. The validation set was used for model internal validation.

#### 2.5.3. Model Establishment

Baseline characteristics in Cohort I and Cohort II were compared using one-way ANOVA for continuous variables and Chi-Squared (χ^2^) tests for categorical variables. Candidate predicting variables were first assessed using univariate logistic regression models in the training dataset. To construct a multivariate logistic prediction model, variables were chosen based on the following criteria: (1) *p* value of less than 0.15 from univariate analyses; (2) collinearity assessment using Pearson’s correlation coefficient with the correlation coefficient lager than 0.80; (3) review of the related literature [13]; and (4) expert opinions from the author groups. The sample size requirement is examined using event per variable [23].

#### 2.5.4. Model Validation

The final multivariate prediction model was then validated internally using the validation set of Cohort I and externally using Cohort II. Discriminative ability was assessed by receiver operating characteristic (ROC) curve analysis and was presented as AUC. AUC values ranged between 0.5 and 1, with a higher value indicating a better discriminative ability. The calibration (goodness-of-fit) was evaluated using the Hosmer–Lemeshow test [24]. After splitting the study population into quintiles, it assesses whether the observed event rates match the expected event rates in each subgroup. *p* values less than 0.05 indicate evidence of poor fit. The overall performance of the model was evaluated using the Nagelkerke R2 and the Brier score [24]. Nagelkerke R2 ranges from 0 to 1, where 0 indicates that the model does not explain any variation, and 1 denotes that it perfectly explains the observed variation. Brier scores, ranging between 0 (perfect) and 0.25 (worthless), verify the accuracy of a probability forecast [25]. The decision curve analysis (DCA) was applied to examine the clinical benefit of the derived model.

#### 2.5.5. Construction and Validation of the UST-CDST

The prediction model was then transformed into a CDST. The final single model equation was transformed into a CDST, and points were assigned to each variable based on multiplication of β coefficient by 10 and rounding to the nearest value. The normality of the continuous variables was examined. If any abnormal distribution was observed, the variable was converted into a categorical value for CDST construction. The cut-off point for low-risk and high-risk groups of CD progression was determined using Cohort I (both the training set and the internal validation set). The top 50% were considered to be at high risk while the bottom 50% were considered to be at low risk of disease progression. The obtained cut-off point was then applied to the external validation cohort (Cohort II), and the performance and generalizability of the CDST were assessed using Pearson’s chi-squared test.

All statistical analyses were conducted using R version 4.3.0. Summary statistics were presented as mean ± standard deviation (SD), median, or frequencies and proportions.

## 3. Results

### 3.1. Patient Characteristics

**Figure 1:**
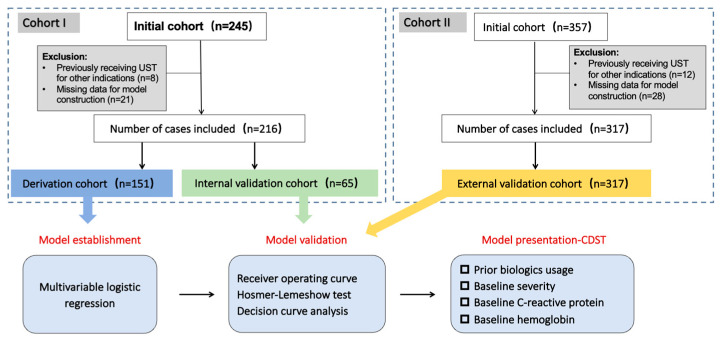
Flowchart of model establishment, validation, and presentation.

Totally, 602 patients were enrolled, of whom 533 were included in the analysis. Among 245 patients with CD in SRRSH-IBC who received UST therapy, 8 patients were excluded due to prior UST exposure for other clinical indications, and another 21 were excluded due to missing data for model construction (Figure 1). A total of 216 patients with CD were finally included (Cohort I). These patients were then randomly allocated into a training set (*n* = 151) and an internal validation set (*n* = 65) through the random split verification. Similarly, 317 out of 357 UST-treated patients with CD from the First Affiliated Hospital and the Sixth Affiliated Hospital of Sun Yat-sen University were included for Cohort II (Figure 1). Comparable event rates (48/216,22.22%; 87/317, 27.44%) were observed between Cohort I and Cohort II. In Cohort I, hospitalization (45 cases), surgery (11 cases), and complications (4 cases) were reported. Due to the low event rates for some outcomes, separate analyses for each event type were not performed.

Baseline characteristics are presented in Table 1. Compared with Cohort II, patients in Cohort I had more severe disease activity according to CDAI (*p* < 0.001), higher proportion of perianal involvement (*p* < 0.001), higher proportion of history of gastrointestinal surgery (*p* < 0.001), lower baseline white blood cell count (*p* < 0.001), and lower baseline platelet count (*p* < 0.001) and were less frequently exposed to prior immunosuppressant therapy (*p* < 0.001). Other characteristics, including disease location, disease behavior, and smoking status, also varied between Cohort I and Cohort II (Table 1).

### 3.2. Variable Selection

As summarized in Table 2, prior biologics usage (OR, 1.58; 95% CI, 0.74–3.39), baseline albumin (OR, 0.90; 95% CI, 0.83–0.96), baseline neutrophil (OR, 0.78; 95% CI, 0.62–0.99), baseline hemoglobin (OR, 0.98; 95% CI, 0.97–1.00), baseline white blood cell (OR,0.80; 95% CI,0.66–0.97), baseline CRP (OR, 1.01; 95% CI, 1.00–1.02), baseline severity (OR, 1.00; 95% CI, 1.00–1.01), and disease behavior were associated with disease progression with *p* values of less than 0.15 in the univariate logistic analysis. As shown in Supplementary Figure S1, white blood cell and neutrophil counts were excluded due to the presence of high collinearity between them (correlation coefficient > 0.80). According to literature review and expert opinions, baseline albumin was excluded while prior biologics usage, baseline disease severity, baseline CRP, and baseline hemoglobin were included in the multivariate analysis. In accordance with the principle of a minimum of 10 events per variable, the number of events in Cohort I (48/216) and Cohort II (87/317) provided adequate sample size for model development and outcome validation [26].

The final logistic regression model for disease progression was as follows:
Logit (Pr (Progression = Y) = −2.2395 (intercept) + [0.3877 when prior biologics exposure is true] + [0.0031 × disease severity as CDAI score] + [0.0069 × baseline CRP in mg/L] − [0.0145 × baseline hemoglobin in g/L]


### 3.3. Model Performance and Validation

**Figure 2:**
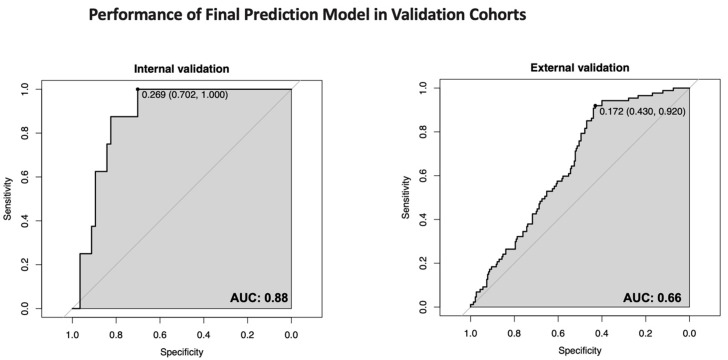
Receiver operating characteristic curves for the UST-CDST to predict the risk of CD progression under UST treatment.

As shown in Figure 2 and Table 3, the AUC of the model was 0.88 (95% CI, 0.78–0.97) in the internal validation cohort and 0.66 (95% CI, 0.60–0.72) in the external validation cohort, demonstrating a moderate discriminative ability. The Hosmer–Lemeshow test χ^2^ equaled 11.34, with a *p* value of 0.18 (>0.05), indicating that the difference between the predicted value and the observed value was not statistically significant. Using the prediction model to guide UST therapy decisions significantly improves net benefits over single or full models within a specific risk threshold range, enhancing clinical outcomes (Supplementary Figure S2). The clinical importance of a history of gastrointestinal surgery in CD management is well-recognized [2]. Nevertheless, its addition to our multivariate model failed to improve predictive utility, underscoring the specificity of our original feature selection (Supplementary Figure S3).

### 3.4. Clinical Decision Support Tool

**Figure 3:**
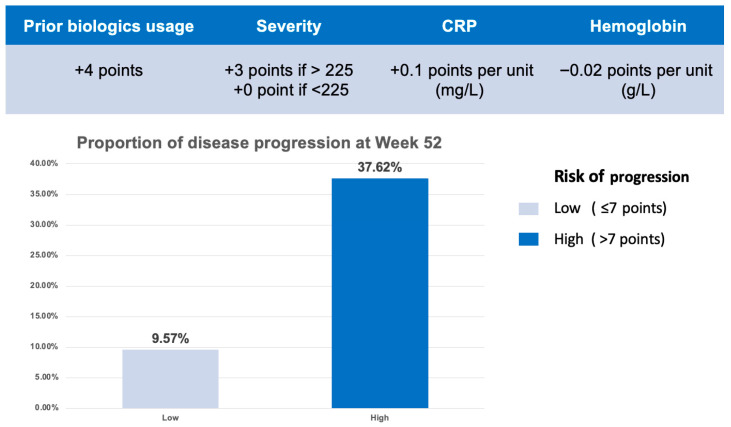
Clinical decision support tool (CDST).

The final model equation was transformed into a CDST to simplify and promote its clinical utility (Figure 3). Points were assigned by multiplying the β coefficient by 10 and rounding to the nearest value. Therefore, 4 points were assigned to individuals with prior biologics usage. Abnormal distribution was observed in baseline disease severity in Cohort I. Thus, it was converted into a categorical variable using the median of CDAI scores (median = 225) with a weight of 3 points in the CDST. Given the normal distribution of baseline CRP, it was considered as a continuous variable in the final CDST with a weight of 0.10 points per unit (mg/L). Similarly, hemoglobin was assigned a weight of −0.02 points per unit (g/L). Five points were added to the final calculation to avoid negative numbers. As described in the methods, a cut-off of 7 points was used to divide patients in Cohort I into distinct groups. When validating in Cohort II, patients in the high-risk group (>7 points) were more likely to experience disease progression following UST therapy compared with those in the low-risk group (≤7 points) (9.57% vs. 37.62%, *p* < 0.05). The cut-off point had a good discriminative ability for identifying patients who were likely to experience disease progression following UST therapy.

## 4. Discussion

In this study, a model presented as a UST-CDST for predicting the risk of disease progression was developed and validated to identify patients with CD who were more likely to benefit from UST therapy. Specifically, four predictors (prior biologics usage, baseline disease severity, baseline CRP, and baseline hemoglobin) were selected to build this model. The AUC of the model was 0.88 for internal validation and 0.66 for external validation, which was comparable to previous studies [16,17]. Finally, the model was transformed into a CDST, which further stratified patients into low-risk and high-risk groups for disease progression with a cut-off value of 7 points.

Based on the magnitude of the β coefficients among four predicting variables, prior biologics usage had the most significant impact on predicting CD-related disease progression in patients treated with UST. Previous studies consistently demonstrated the considerable impact of prior biologics exposure on the efficacy of current medications [27,28,29]. In a recent report, patients with CD without a history of biologics failure or intolerance had a cumulative clinical remission rate of 55.5% at week 16 after UST induction, while patients with a history of biologic failure or intolerance had a corresponding cumulative rate of 24.1% (*p* < 0.001) [29]. However, our study did not conduct a detailed classification of prior biologics when selecting predictive variables. Some patients may have experienced treatment failure with more than one biological agent before UST initiation, while some of them may have failed treatment with either a TNF antagonist or VDZ. A more detailed classification of prior biologics may provide a more precise prediction of drug efficacy [6].

CRP is an acute-phase protein that reacts with the C-polysaccharide of *Streptococcus pneumonia* [30]. It is commonly used in the evaluation of many inflammatory diseases and functions as an effective monitoring biomarker to assess patients in the active phase of IBD [31]. As an easy and non-invasive approach, CRP measurement can be included in the routine management of IBD and added conveniently to a CDST [32]. Some previous CDSTs of VDZ therapy also included CRP as a predicting variable for drug efficacy [5,33]. However, CRP was not consistently selected for CDST construction for UST [16,17]. The difference in variable selection approaches may have contributed to the variability in prediction models [34]. Similarly to CRP, hemoglobin levels were found useful in detecting colorectal diseases while few studies included hemoglobin in the prediction models of disease progression [5,35,36]. A retrospective multicenter study involving 439 patients with CD receiving UST identified that higher baseline hemoglobin levels were associated with a higher likelihood of achieving remission, which was consistent with our current finding [1].

CDAI, widely used in both clinical practice and scientific research, is a well-recognized index for evaluating CD activity and drug efficacy [19]. In this study, both the univariate and multivariate analyses suggested that higher baseline CDAI was associated with higher probability of disease progression. A prospective multicenter study of 114 adult patients with active CD initiating UST demonstrated that baseline Harvey–Bradshaw Index (HBI) scores, another measurement of CD activity, independently predicted remission at both weeks 52 and 104, with a higher HBI score at baseline associated with a lower probability of long-term remission [37]. Collectively, these findings suggest that UST may have improved effectiveness among patients with mild to moderate disease courses.

To our knowledge, this is the first study to establish a UST-CDST for predicting CD progression in the Chinese population using real-world data. The model has been validated using a large independent cohort, and its discriminative ability has been confirmed by the relatively high AUC values [5,17,38]. There were several strengths of our study. First, given that UST was approved for the treatment of CD in China later than in Western countries, our sample size was comparable or superior to similar studies conducted in the Chinese population [39]. Second, all three large referral hospitals supporting this study have set up officially registered and well-managed IBD centers, thus ensuring the quality of data and the representativeness of patients with IBD in China. Third, the four variables included in the UST-CDST are routine and easily accessible clinical measures. The simplicity of the UST-CDST has enhanced its generalizability. Fourth, we chose a common time point to evaluate the efficacy of UST treatment in clinical practice, thus providing a more rational and practical tool for the study population [22].

Although the overall study design is retrospective, data from Cohort I, which included both the derivation and the internal validation datasets, have been prospectively collected and regularly updated since 2020. Consequently, the level of evidence in this study is, to some extent, comparable to that of prospective studies. Comparing our CDST to previous CDSTs (e.g., VDZ-CDST) aiming to optimize treatment strategies in Crohn’s disease through predictive modelling, we differ fundamentally in scope and clinical purpose. The VDZ-CDST is designed to predict treatment response, thereby assisting in patient selection for that specific drug. In contrast, our model focuses on predicting disease progression, defined using 3 correlated conditions: CD-related surgery, CD-related hospitalization, and CD-related complications. Our tool potentially helps in risk stratification and implementing intensive monitoring strategies for high-risk patients after the treatment choice is made. While previous studies on CDST of VDZ therapy have combined common predictors of related outcomes to prevent uncertainty in interpretation and potentially diverging predictions [5], a separate analysis of the aforementioned 3 conditions may provide additional insights. However, it was hindered by the current sample size. Enlarging the sample size and refining the classification of outcomes might enable our UST-CDST to provide more comprehensive and accurate suggestions on clinical decision. To our knowledge, biologic agents like UST show much reduced effectiveness in patients with CD with fibrous stenosis compared to those with inflammatory stenosis [40]. The present lack of differentiation of stenosis types may have limited the predictive effect of our model, while a more detailed classification may help to improve the diagnostic performance. Another limitation of our study is that we did not systematically assess clinical relapse events that required corticosteroid therapy within the follow-up period. The absence of this data precludes a more comprehensive comparison of the long-term treatment effectiveness between groups, which should be explicitly evaluated in future studies. Our UST-CDST was developed and validated specifically for patients treated with UST. The predictors and their corresponding weights are tailored to this therapeutic context and are not directly applicable to patients receiving other medications. Further research is needed to develop and validate similar tools for other treatment regimens.

In summary, we developed and validated a clinical prediction model for disease progression in Chinese population with CD. The model was subsequently transformed into a scoring system, UST-CDST, to stratify patients with UST treatment into different risk groups. Prospective use of the UST-CDST may streamline therapeutic decision-making, promote precision medicine, and enhance the cost-effectiveness of UST in CD, by identifying patients who are more likely to benefit from UST. Further research is warranted to further optimize the predictive accuracy of the UST-CDST and to comprehensively evaluate its performance across diverse clinical settings and ethnic populations.

## Figures and Tables

**Table 1:** Baseline characteristics of patients from internal and external cohorts.

Characteristics	Internal (*n* = 216)	External (*n* = 317)	*p* Value
Male, n (%)	96 (44.44)	76 (23.97)	0.072
Median age at diagnosis (IQR), years	31.35 ± 12.06	27.80 ± 11.46	0.118
Median age at first UST (IQR), years	35.60 ± 12.70	30.00 ± 11.98	0.022
BMI (IQR), kg/m^2^	19.28 ± 2.90	19.71 ± 3.11	0.112
Smoking status, n (%)			<0.001
Never	196 (90.74)	280 (88.33)	
Current smoker	11 (5.10)	35 (11.04)	
Smoked previously	9 (4.16)	2 (0.63)	
Median interval from onset to diagnosis, years	2.41 ± 3.39	3.33 ± 3.77	0.004
Severity (CDAI score)	211.55 ± 62.96	151.89 ± 103.36	<0.001
Location, n (%)			<0.001
Ileal	67 (31.02)	85 (26.82)	
Colonic	3 (1.39)	18 (5.68)	
Ileocolonic	91 (42.13)	184 (58.04)	
Upper GI	55 (25.46)	30 (9.46)	
Behavior, n (%)			<0.001
Nonstricturing, nonpenetrating	72 (33.33)	164 (51.74)	
Stricturing	97 (44.91)	96 (30.28)	
Penetrating	47 (21.76)	57 (17.98)	
Perianal involvement, n (%)	147 (71.36)	164 (51.74)	<0.001
EIMs, n (%)	44 (20.37)	42 (13.25)	0.028
Prior 5-ASA usage, n (%)	95 (43.98)	173 (54.57)	0.016
Prior steroid usage, n (%)	52 (24.07)	113 (35.65)	0.004
Prior immunosuppressant usage, n (%)	91 (42.12)	182 (57.41)	<0.001
Prior biologics usage, n (%)	126 (58.33)	172 (54.26)	0.35
History of gastrointestinal surgery, n (%)	96 (44.44)	80 (25.24)	<0.001
History of perianal surgery, n (%)	80 (37.04)	111 (35.02)	0.633
C-reactive protein, mg/L	16.39 ± 26.72	18.61 ± 27.47	0.346
Erythrocyte sedimentation rate, mm/h	18.47 ± 17.22	23.35 ± 23.50	0.008
White blood cell, 10^9^/L	5.97 ± 2.13	6.72 ± 2.47	<0.001
Neutrophil, 10^9^/L	3.93 ± 1.74	4.17 ± 1.82	0.113
Blood platelet count, 10^9^/L	259.74 ± 22.35	306.57 ± 103.83	<0.001
Hemoglobin, g/L	126.67 ± 22.35	123.22 ± 21.51	0.072
Albumin, g/L	39.02 ± 5.02	39.40 ± 6.17	0.460
Concomitant drug usage, n (%)	17 (7.87)	37 (11.88)	0.154

IQR, interquartile range; UST, Ustekinumab; BMI, body mass index; CDAI, Crohn’s Disease Activity Index; GI, gastrointestinal; EIMs, extra-intestinal manifestations; 5-ASA, 5-aminosalicylic acid.

**Table 2:** Univariate and multivariable analyses of factors predicting disease progression.

Characteristics	Univariate Analysis		Multivariate Analysis	
	OR (95% CI)	*p* Value	OR (95% CI)	*p* Value
Sex	0.66 (0.3–1.43)	0.29		
Median age at diagnosis	1.01 (0.98–1.04)	0.36		
Median age at first UST	1.02 (0.99–1.04)	0.25		
BMI	0.97 (0.86–1.10)	0.68		
Smoking status				
Never	(reference)			
Current smoker	0.70 (0.14–3.46)	0.66		
Smoked previously	1.68 (0.38–7.40)	0.49		
Severity (CDAI score)	1.00 (1.00–1.01)	0.10	1.00 (0.99–1.01)	0.30
Median interval from onset to diagnosis, years	1.01 (0.92–1.12)	0.79		
Location				
Ileal	(reference)			
Colonic	2.25 (0.18–27.96)	0.53		
Ileocolonic	1.97 (0.78–4.95)	0.15		
Upper GI	1.80 (0.62–5.20)	0.28		
Behavior				
Nonstricturing, nonpenetrating	(reference)			
Stricturing	2.29 (0.92–5.72)	0.28		
Penetrating	2.45 (0.86–6.96)	0.08		
Perianal involvement	0.86 (0.39–1.91)	0.72		
EIMs	0.71 (0.27–1.91)	0.50		
Prior 5-ASA usage	0.76 (0.36–1.59)	0.47		
Prior steroid usage	1.15 (0.49–2.66)	0.75		
Prior immunosuppressant usage	1.23 (0.60–2.55)	0.57		
Prior biologics usage	1.58 (0.74–3.39)	0.14	1.47 (0.67–3.34)	0.30
History of gastrointestinal surgery	1.47 (0.71–3.03)	0.30		
History of perianal surgery	1.36 (0.65–2.85)	0.41		
Concomitant drug usage	1.43 (0.41–5.04)	0.58		
C-reactive protein (mg/L)	1.01 (1.00–1.02)	0.13	1.01 (0.99–1.02)	0.30
Erythrocyte sedimentation rate	1.00 (0.98–1.02)	0.83		
White blood cell	0.80 (0.66–0.97)	0.03		
Hemoglobin (g/L)	0.98 (0.97–1.00)	0.03	0.99 (0.97–1.00)	0.07
Blood platelet count	1.00 (0.99–1.00)	0.63		
Neutrophil	0.78 (0.62–0.99)	0.05		
Albumin	0.90 (0.83–0.96)	0.01		

OR, odds ratio; CI, confidence interval; IQR, interquartile range; UST, Ustekinumab; BMI, body mass index; CDAI, Crohn’s Disease Activity Index; GI, gastrointestinal; EIMs, extra-intestinal manifestations; 5-ASA, 5-aminosalicylic acid.

**Table 3:** Measurements of the model performance.

Characteristics	Final Model
Nagelkerke R2	0.08
Brier score	0.11
ROC-AUC (95% CI) internal validation	0.88 (0.78–0.97)
ROC-AUC (95% CI) external validation	0.66 (0.60–0.72)

ROC, receiver operating characteristic; AUC, area under the curve; CI, confidence interval.

## Data Availability

The data and materials used in this study are not publicly available due to ethical restrictions. Requests should be directed to the corresponding author.

## Supplementary Materials

The following supporting information can be downloaded at https://www.mdpi.com/article/10.3390/jcm14227919/s1. Supplementary Figure S1: Correlation matrix of baseline characteristics with *p* values less than 0.15 in the univariate logistic analysis. Supplementary Figure S2: Decision-curve analysis from the derivation cohort and the internal validation cohort. The blue dashed line represents the simple model, and the red line represents the multivariable logistic regression model. Supplementary Figure S3: Receiver operating characteristic curve for the UST-CDST (adding GI surgery) to predict the risk of CD progression under UST treatment.

## Abbreviations

The following abbreviations are used in this manuscript:

IBD	Inflammatory bowel disease
CD	Crohn’s disease
UST	Ustekinumab
CDST	Clinical decision support tool
AUC	Area under the curve
