# Supplementary figures and images for: Development and Validation of a Clinical Decision Support Tool to Predict Disease Progression in Crohn’s Disease Treated with Ustekinumab
Source: J Clin Med. 2025 Nov 8;14(22):7919. doi: 10.3390/jcm14227919 (PMC12653264; doi:10.3390/jcm14227919)

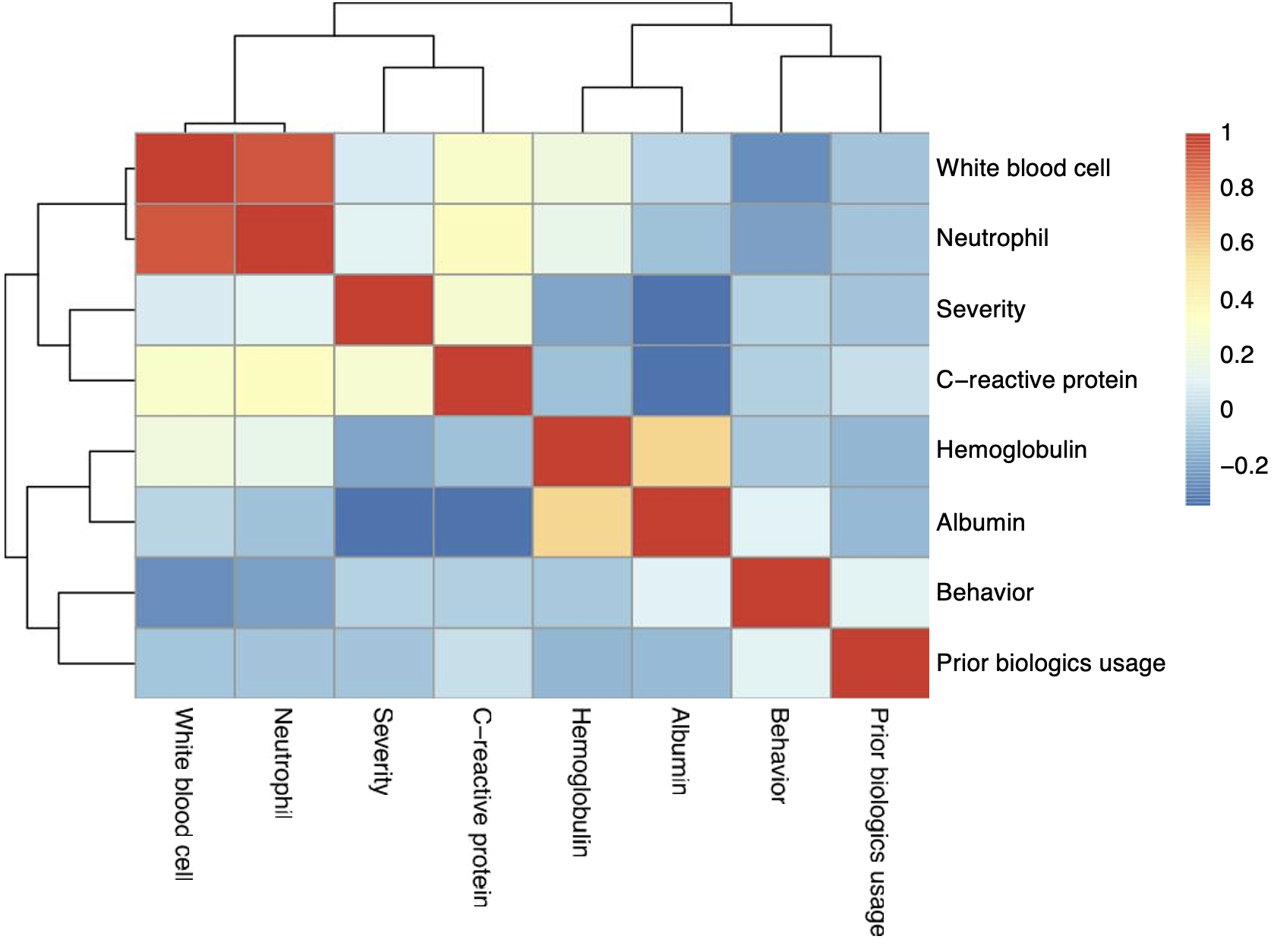

Supplement: Supplementary file 1 [file jcm-14-07919-s001.zip › Figure S1.tiff]

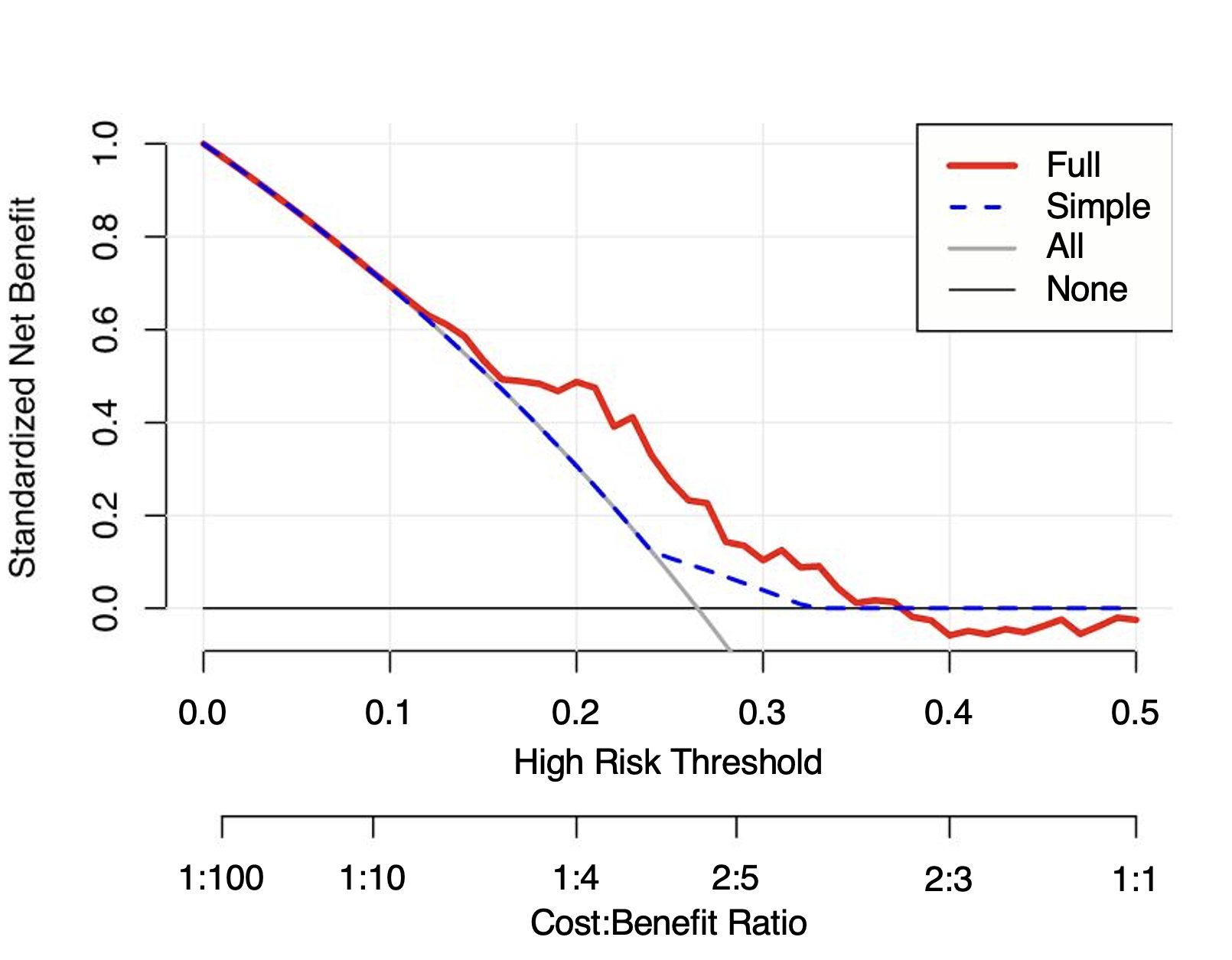

Supplement: Supplementary file 1 [file jcm-14-07919-s001.zip › Figure S2.tiff]

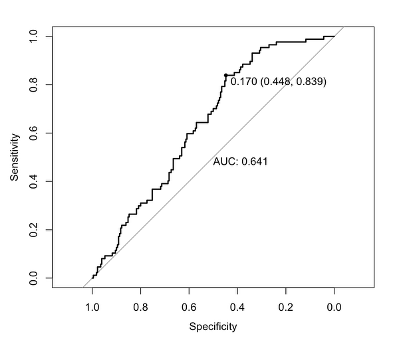

Supplement: Supplementary file 1 [file jcm-14-07919-s001.zip › Figure S3.png]
